# Percutaneous Endoscopic Transforaminal Discectomy versus Conventional Open Lumbar Discectomy for Upper Lumbar Disc Herniation: A Comparative Cohort Study

**DOI:** 10.1155/2020/1852070

**Published:** 2020-03-02

**Authors:** Ziquan Li, Cong Zhang, Weisheng Chen, Shugang Li, Bin Yu, Hong Zhao, Jianxiong Shen, Jianguo Zhang, Yipeng Wang, Keyi Yu

**Affiliations:** ^1^Department of Orthopedic Surgery, Peking Union Medical College Hospital, Peking Union Medical College and Chinese Academy of Medical Sciences, Beijing 100730, China; ^2^Department of Endocrinology, China-Japan Friendship Hospital, Beijing 100029, China; ^3^Department of Orthopedic Surgery, Shanghai Jiao Tong University Affiliated Sixth People's Hospital, Shanghai Jiao Tong University, Shanghai 200233, China

## Abstract

**Background:**

Percutaneous endoscopic transforaminal discectomy (PETD) is regarded as a viable alternative option for upper lumbar disc herniation (LDH). However, few studies have evaluated PETD for upper LDH, and no study has compared the advantages of endoscopic procedures versus conventional surgery. The present study was aimed at comparing the surgical outcome and safety of PETD versus conventional open lumbar discectomy in the treatment of upper LDH.

**Methods:**

Data from 42 patients treated for upper LDH from July 2015 to July 2018 were retrospectively analyzed, including 21 patients treated with PETD (PETD group) and 21 patients treated with conventional posterior lumbar discectomy (open group). The two groups were compared regarding demographic information, physical examination, radiological evaluations, and perioperative indicators. The clinical outcomes were assessed in accordance with the Oswestry Disability Index (ODI), visual analog scale (VAS), and modified MacNab criteria.

**Results:**

The postoperative ODI and VAS scores were significantly improved in both groups compared with the preoperative baseline values (*P* < 0.001), and the satisfactory rate was 90.5% in both groups in accordance with the modified MacNab criteria. There were no significant differences between the two groups in the clinical outcomes and complication rate (*P* < 0.001), and the satisfactory rate was 90.5% in both groups in accordance with the modified MacNab criteria. There were no significant differences between the two groups in the clinical outcomes and complication rate (*P* < 0.001), and the satisfactory rate was 90.5% in both groups in accordance with the modified MacNab criteria. There were no significant differences between the two groups in the clinical outcomes and complication rate (

**Conclusions:**

PETD has a similar outcome to the conventional surgical method for the treatment of upper LDH but provides the typical advantages of minimally invasive procedures such as reduced iatrogenic injury, minimal activity restrictions, and accelerated ambulation recovery postoperatively.

## 1. Introduction

Upper lumbar disc herniation (LDH) refers to the rupture of the fibrous annulus and protrusion of the nucleus pulposus at L3-4 or above and has a low incidence of 1–10.4% but a high rate of misdiagnosis [1–3]. Compared with lower LDH, disc herniation in the upper lumbar spine involves unique anatomic characteristics, including a small spinal canal, narrow distance between the exiting nerve root and the dura, short nerve roots, and a location adjacent to the lumbosacral enlargement of the spinal cord [4]. Thus, it is more essential to perform surgical decompression for upper LDH than lower LDH, although the challenges and surgical risks are higher and the surgical outcome is less satisfactory [5, 6].

In recent years, increasing numbers of clinical studies have confirmed that percutaneous endoscopic lumbar discectomy has similar effectiveness to conventional surgery but has the advantages of less blood loss, decreased soft tissue damage, and shorter postoperative recovery time [7]. With the development and advancement of surgical techniques, the application of the percutaneous spinal endoscopic technique is expanding [8–10]. Percutaneous endoscopic transforaminal discectomy (PETD) is reportedly a viable alternative option for upper LDH that does not require laminectomy and dural traction [6, 11, 12]. However, related articles about PETD for upper LDH are limited, and no study has compared PETD and conventional open discectomy in treating upper LDH. Therefore, we performed a retrospective comparative study of PETD versus conventional open discectomy to evaluate the surgical outcomes and advantages of each technique and to describe the technical strategies specific to PETD for upper LDH.

## 2. Methods

### 2.1. Cohort Collection

We recruited 42 consecutive Chinese patients diagnosed with symptomatic upper LDH from July 2015 to July 2018 at Peking Union Medical College Hospital (PUMCH). The inclusion criteria were (1) a single segment of central, paracentral, or prolapsed upper LDH demonstrated on computed tomography and magnetic resonance imaging, (2) unilateral radicular leg pain consistent with the radiographic findings and failure of extensive conservative therapies for more than 3 months, including medications, physiotherapy, and other treatments, and (3) no segmental instability on plain radiography. The exclusion criteria were the presence of recurrent disc herniation after prior surgery, severe central spinal stenosis, tumor or tuberculosis or pyogenic discitis, intervertebral disc calcification, painless motor weakness, and cauda equina syndrome.

Demographic information, physical examination findings, clinical symptoms, and a detailed medical history were obtained. Each patient underwent radiological evaluations including lumbar anterior-posterior (AP), lateral neutral, and dynamic position plain radiographs, computed tomography, and magnetic resonance imaging. The surgical technique was selected based on the surgeons' preferences. PETD was performed in 21 patients (PETD group), while another 21 patients underwent posterior lumbar discectomy and internal fixation with the conventional technique (open group).

Each patient provided written informed consent prior to study participation. The study was approved by the Department of Scientific Research and Ethics Committee of PUMCH in China.

### 2.2. Surgical Procedures

**Figure 1:**
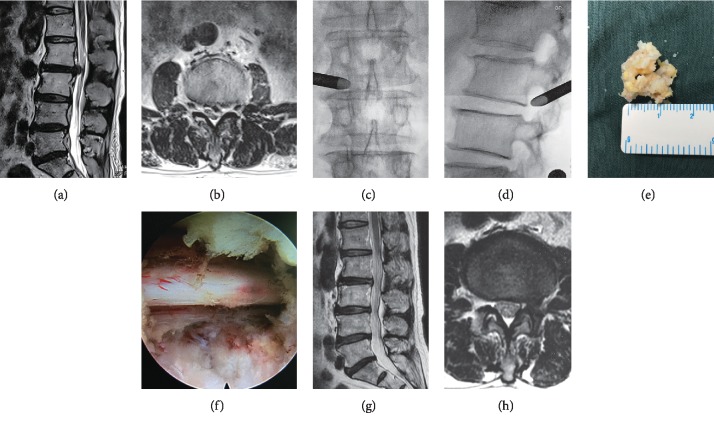
Images from a typical case of percutaneous endoscopic transforaminal discectomy in a 73-year-old male with upper lumbar disc herniation at L2–3. (a, b) Preoperative sagittal and axial T2-weighted magnetic resonance imaging (MRI) shows lumbar disc herniation at L2–3. (c, d) Anteroposterior and lateral fluoroscopic views depict the working cannula positioned at the foraminal area at L2–3. (e, f) Removal of the herniated fragment and intraoperative view of the nerve root after decompression. (g, h) At 6-month follow-up, postoperative sagittal and axial T2-weighted MRI illustrates complete excision of the prolapsed disc, without recurrence, or residual disc at L2–3.

PETD was performed with the patient in the lateral decubitus position under local anesthesia. The surgical segment and puncture needle entry point were confirmed under AP and lateral C-arm fluoroscopic guidance. A steep trajectory angle (35–45°) of the needle and continuous feedback from patients were considered to avoid injuring the dural sac and traversing nerve roots. The needle was positioned at the posterior edge of the intervertebral disc and the vertebral body on lateral fluoroscopy when it approached the middle pedicular line on the AP fluoroscopic view. The needle was then replaced with a guidewire, a dilating obturator was passed over the guidewire, and a working cannula (joimax endoscopic system, TESSYS, Germany) was inserted. The ruptured fragments of the herniated disc were endoscopically resected with forceps and a bipolar radiofrequency coagulator (Elliquence, New York, USA). Attention was paid to the space between the disc and the ligamentum flavum and to the ventral and lateral sides of the traversing nerve root to ensure that adequate decompression was achieved. At the end of the operation, the surgeon confirmed the following endoscopic decompression criteria: free mobilization of the neural tissue, independent pulsation of the dural sac and nerve root (consistent with the heart rate), recovery of the anatomical position of the neural tissue, and improvement of the blood supply to the neural tissue. The surgeon also ensured that the symptoms were alleviated and the intraoperative straight leg raising test was negative.  Figure 1 shows images from a typical patient with upper LDH who underwent successful PETD and close follow-up.

Conventional open lumbar discectomy was performed via the posterior approach. The epidural space was exposed through a midline incision after adequate detachment of the paravertebral muscles, laminectomy, and ligamentum flavum resection. Partial facetectomy was performed on the symptomatic side, and then, the herniated disc was removed while the spinal cord and nerve root were being protected. Internal fixation and bone graft fusion were performed with or without interbody fusion. The operation was finished with hemostasis, irrigation, epidural drainage, and wound closure.

### 2.3. Clinical Assessments and Follow-Up

A follow-up was performed via telephone or clinical visits at 6 weeks, 3 months, 6 months, and 1 year postoperatively. Subsequently, the follow-up was performed every 1 to 3 years, depending on the patient's course of recovery.

The Oswestry Disability Index (ODI) scores and visual analog scale (VAS) scores for lower back pain and sciatica were recorded preoperatively, postoperatively, and at the final follow-up. The clinical outcomes were evaluated based on the modified MacNab score. Perioperative indicators such as the duration of surgery, estimated blood loss, would drainage volume, blood transfusion, and postoperative hospital stay were compared between the two groups. Surgical complications and recurrence were also recorded.

### 2.4. Statistical Analysis

The relevant features were compared using the independent sample *t*-test, while Fisher's exact test was used for categorical variables. Results are expressed as mean ± standard deviation, and *P* values of <0.05 were considered statistically significant.

## 3. Results

### 3.1. Demographic and Clinical Information

The PETD group comprised 21 patients diagnosed with upper LDH, including 13 men and eight women, with a mean age of 49.8 ± 17.9 years (range, 16–75 years). The mean symptom duration in the PETD group was 8.5 ± 9.6 months. The preoperative clinical signs in the PETD group were motor weakness in 10 patients (47.6%), positive Lasègue sign in 10 patients (47.6%), positive Bragard sign in eight patients (38.1%), and lower limb paresthesia in seven patients (33.3%). Disc herniation occurred at L1–2, L2–3, and L3–4 in 4.8%, 33.3%, and 61.9% of the patients in the PETD group, respectively. There were no significant differences between the PETD group and the open group regarding age, sex, duration of symptoms, clinical signs, and operative level. The detailed demographic and clinical information of the two groups is presented in Table 1.

### 3.2. Perioperative Parameters and Complications

All 42 patients underwent successful single-level upper LDH surgery and were followed-up for 12 to 48 months (mean, 34.1 months).  Table 2 summarizes the parameters related to the operative procedures, such as operation time, intraoperative blood loss, drainage volume, and hospitalization time. Compared with the open group, the PETD group had significantly smaller volumes of bleeding and postoperative drainage and a significantly shorter surgical duration and postoperative hospitalization (*P* < 0.001). Additionally, three patients in the open group received blood transfusions because of intraoperative blood loss and postoperative anemia.

No patient in either group had nerve root injury, fragment omissions, recurrent disc herniation, or cardiac or cerebrovascular complications. In the open group, there were no complications related to internal fixation such as breakage, looseness, or displacement. Complications in the open group included poor wound healing in two patients and deep vein thrombosis in one patient. In the PETD group, two patients had a dural tear with cerebrospinal fluid leakage during surgery; these two patients rest in bed in the supine position until wound drainage removal on postoperative day 2 and recovered well. Furthermore, one patient in each group experienced postoperative dysesthesia with transient lower limb weakness because of irritation of the exiting nerve root. All complications were improved after conservative treatments without revision surgery. The complication rate did not significantly differ between the two groups (*P* = 0.697) (Table 2).

### 3.3. Therapeutic Effects

Both groups showed significant improvements in the VAS scores for lower back pain and sciatica at the final follow-up in comparison with the preoperative baseline values (*P* < 0.001), and the VAS scores at the final follow-up did not significantly differ between the two groups (*P* > 0.05). The mean ODI scores improved from 63.8 ± 18.3% to 12.0 ± 6.8% in the PETD group and from 59.3 ± 15.7% to 15.9 ± 6.7% in the open group; the ODI scores at the final follow-up did not significantly differ between the two groups (*P* > 0.05).

In accordance with the modified MacNab scores, the outcome in the PETD group was excellent in 11 cases, good in eight, and fair in two, giving an excellence or good rate of 90.5%. In the open group, the outcome was excellent in nine cases, good in 10, fair in one, and poor in one. The distribution of the MacNab criteria assessments did not significantly differ between the two groups (*P* = 0.719) (Table 3).

## 4. Discussion

Anatomically, the upper lumbar spine consists of a narrower spinal canal and a larger dural sac than the lower lumbar spine, with the lumbar nerve roots and cauda equinus presented together. Thus, both of these structures may be simultaneously compressed and disordered by a protrusive upper lumbar disc [13, 14]. As the nerve roots in the upper lumbar spine do not innervate any specific muscles, upper LDH results in nonspecific clinical symptoms and neurological findings, which can lead to missed diagnosis of upper LDH [15, 16].

As upper LDH has a low incidence, anatomical complexity, and high misdiagnosis rate, the surgical outcome of upper LDH is less satisfactory than that of lower LDH. An excellent or good surgical outcome of upper LDH has been reported in 81% of 41 patients [17], 78% of 45 patients [6], and 80% of 141 patients [18]. Currently, upper LDH is treated via several anterior and posterior approaches and various techniques [19–21]. The conventional posterior approach enables full decompression of the spinal canal and nerve root but requires a wide laminectomy and facetectomy to obtain adequate bony exposure; however, upper lumbar discectomy can be performed safely, avoiding injury and overretraction of the neural tissue. The disadvantage of the conventional posterior approach is that patients must undergo internal fixation and lumbar fusion, as the excessive removal of bony tissue may induce iatrogenic spondylolysis and segmental spinal instability [22].

To avoid the iatrogenic instability and spinal fusion resulting from conventional posterior lumbar discectomy for upper LDH, minimally invasive percutaneous endoscopic transforaminal surgery that was previously used for lower LDH has become an alternative technique for treating upper LDH; compared with the conventional approach, PETD for upper LDH reportedly results in decreased iatrogenic injury, accelerated rehabilitation, and reduced hospitalization. In the present study, the outcome in accordance with the MacNab criteria was excellent/good in 90.5% of patients in the PETD group. The ODI and VAS scores for lower back pain and sciatica at the final follow-up were significantly improved in both the PETD and open groups and did not significantly differ between groups. Therefore, PETD achieved a similar effect to the conventional surgical method but significantly reduced the operation time, blood loss, blood transfusion rate, postoperative hospitalization time, and incidence of wound complications.

The distinctive advantages of PETD over conventional posterior lumbar discectomy may depend on the following factors [23, 24]. First, PETD results in shorter operative duration, minimal blood loss and wound drainage, less wound complications and postoperative instability due to the reduced iatrogenic tissue trauma resulting from the small skin incision, less paravertebral muscle injury, and preservation of posterior ligamentous and bony structures. Second, PETD is feasible under local anesthesia combined with conscious sedation, contributing to less anesthesia-related complications and quicker recovery with a shorter inpatient stay. The early rapid recovery has been shown to be effective at reducing deep vein thrombosis. Furthermore, using the transforaminal endoscopic approach at the upper lumbar level enables the extruded disc to be removed without dural retraction, and the segmental motion can be preserved. In consequence, unnecessary application of an implant could be reduced by PETD for the treatment of upper LDH.

There was no significant difference between the PETD and open groups regarding common postoperative complications such as fragment omissions, postoperative dysesthesia, and recurrent disc herniation. However, two patients in the PETD group experienced a dural tear with cerebrospinal fluid leakage (9.5%); one of the patients developed a ventral dural tear during the separation of adhesions between the intervertebral disc and the posterior longitudinal ligament, while the other patient incurred an intraoperative dural tear due to foraminoplasty with trephine for foraminal stenosis. The reported incidence of dural injury in PETD is 0.1–3.7% [25, 26], which is lower than that in our study on patients with upper LDH.

Although most dural tears occur during the pursuit of more definite decompression and clearer visualization of the decompressed neural tissues [27], the possible reasons for dural tears are mechanical tearing caused by surgical tools or thermal injury caused by the bipolar radiofrequency coagulator. The following technical points of PETD should be considered to prevent dural tear and nerve root injury in the treatment of upper LDH. Firstly, a steep approach (needle trajectory of 35–45°) and lateral landing are recommended for PETD at upper lumbar levels [27]. A steeper trajectory angle and working cannula laterally located at the middle pedicular line on an AP fluoroscopic view are able to guarantee an adequate working space without neural damage, as the upper lumbar discs are more concave and the facets are oriented more parallel to the midsagittal plane compared with the lower lumbar discs [28]. Furthermore, the whole hernia fragment in both the epidural and intradiscal spaces should be completely removed to prevent recurrence. Secondly, unlike in the lower lumbar levels, the neural foraminal zone in the upper lumbar levels is relatively large so it is rare for foraminal stenosis to interfere with the transforaminal approach [29, 30]. Thus, the dural sac is readily exposed through the foraminal window, and preoperative evaluation can prevent the performance of unnecessary foraminoplasty. Endoscopic lateral recess decompression should be considered if foraminoplasty is necessary. Moreover, dural tear is more likely to occur during PETD when the patient has degenerative scoliosis or severe adhesion of the nerve root, dura mater, intervertebral disc, and posterior longitudinal ligament. Therefore, the adhesions should be carefully separated before the herniated disc is removed; this separation should start with the mild adhesions and progress to the severe adhesions.

## 5. Conclusions

This is the first comparative study of PETD versus conventional open lumbar discectomy for the treatment of upper LDH. We conclude that PETD achieves satisfactory surgical outcomes in the treatment of upper LDH and results in a reduced incidence of iatrogenic injury, minimal activity restrictions, and accelerated ambulation recovery compared with conventional surgical methods.

## Figures and Tables

**Table 1:** Demographic and clinical information of the two groups.

Parameter	PETD group	Open group	*P* value
Number of patients	21	21	
Average age (yrs)	49.8 ± 17.9	49.5 ± 12.6	0.943
Age range (yrs)	16-75	26-66	
Sex (male/female)	13/8	15/6	0.744
Operative level			0.529
L1–L2	1	2	
L2–L3	7	6	
L3–L4	13	13	
Duration of symptoms (months)	8.5 ± 9.6	8.1 ± 8.0	0.892
Clinical signs			0.750
Lasègue sign +	10	13	
Bragard sign +	8	12	
Paresthesia in lower limbs	7	8	
Lower extremity weakness	10	9	

PETD group: patients with upper lumbar disc herniation who underwent percutaneous endoscopic transforaminal discectomy (*n* = 21); open group: patients with upper lumbar disc herniation who underwent conventional posterior lumbar discectomy and internal fixation (*n* = 21).

**Table 2:** Operation parameters and complications of the two groups.

Parameter	PETD group	Open group	*P* value
Operation time (min)	94.5 ± 23.9	148.1 ± 33.2	<0.001
Estimated blood loss (ml)	18.1 ± 9.7	308.6 ± 240.7	<0.001
Drainage (ml)	42.0 ± 78.4	185.0 ± 98.3	<0.001
Blood transfusion	0	3	<0.001
Postoperative hospitalization stay	3.5 ± 1.6	7.7 ± 4.0	<0.001
Complications			0.697
Recurrent disc herniation	0	0	
Cerebrospinal fluid leak	2	1	
Postoperative dysesthesia	1	1	
Deep vein thrombosis	0	1	
Poor wound healing	0	2	

PETD group: patients with upper lumbar disc herniation who underwent percutaneous endoscopic transforaminal discectomy (*n* = 21); open group: patients with upper lumbar disc herniation who underwent conventional posterior lumbar discectomy and internal fixation (*n* = 21).

**Table 3:** Therapeutic effects and modified MacNab criterion assessments of the two groups.

	PETD group	Open group	*P* value
VAS (lower back pain)			
Preoperative	6.0 ± 2.0	5.9 ± 1.7	0.810
Final follow-up	1.4 ± 0.9	1.8 ± 0.7	0.139
VAS (sciatica)			
Preoperative	7.3 ± 1.4	7.1 ± 1.4	0.603
Final follow-up	1.5 ± 1.3	1.3 ± 0.7	0.484
ODI scores			
Preoperative	63.8% ± 18.3%	59.3% ± 15.7%	0.406
Final follow-up	12.0% ± 6.8%	15.9% ± 6.7%	0.080
Modified MacNab			0.719
Excellence	11	9	
Good	8	10	
Fair	2	1	
Poor	0	1	

PETD group: patients with upper lumbar disc herniation who underwent percutaneous endoscopic transforaminal discectomy (*n* = 21); open group: patients with upper lumbar disc herniation who underwent conventional posterior lumbar discectomy and internal fixation (*n* = 21).

## Data Availability

The data used to support the findings of this study are available from the corresponding authors upon request.
